# The Effect of Diabetes Medication on Cognitive Function: Evidence from the PATH Through Life Study

**DOI:** 10.1155/2016/7208429

**Published:** 2016-04-19

**Authors:** Pushpani M. Herath, Nicolas Cherbuin, Ranmalee Eramudugolla, Kaarin J. Anstey

**Affiliations:** Centre for Research in Ageing Health and Wellbeing, Australian National University, Canberra, ACT 0200, Australia

## Abstract

*Objective*. To examine the effect of diabetes treatment on change of measures of specific cognitive domains over 4 years.* Research Design and Methods*. The sample was drawn from a population-based cohort study in Australia (the PATH Through Life Study) and comprised 1814 individuals aged 65–69 years at first measurement, of whom 211 were diagnosed with diabetes. Cognitive function was measured using 10 neuropsychological tests. The effect of type of diabetes treatment (diet, oral hypoglycemic agents, and insulin) on measures of specific cognitive domains was assessed using Generalized Linear Models adjusted for age, sex, education, smoking, physical activity level, BMI, and hypertension.* Results*. Comparison of cognitive function between diabetes treatment groups showed no significant effect of type of pharmacological treatment on cognitive function compared to diet only group or no diabetes group. Of those on oral hypoglycaemic treatment only, participants who used metformin alone had better cognitive function at baseline for the domains of verbal learning, working memory, and executive function compared to participants on other forms of diabetic treatment.* Conclusion*. This study did not observe significant effect from type of pharmacological treatment for diabetes on cognitive function except that participants who only used metformin showed significant protective effect from metformin on domain of verbal learning, working memory, and executive function.

## 1. Introduction

Diabetes is a common and complex metabolic disease that can lead to end-organ damage in almost all vital organs, including the brain. Cognitive decline and dementia are now recognized and investigated as diabetes related complications. Relation between diabetes and cognition may be due to diabetes specific variables (macrovascular and microvascular changes, impaired glucose metabolism, chronic inflammation, hyperinsulinemia, insulin resistance, or oxidative stress), cardiovascular risk factors (hypertension, coronary artery disease), and life style risk factors (smoking, level of physical activity, and diet) [1–4].

Diabetes treatments including oral hypoglycemic agents (OHAs) and insulin that minimize disease symptoms and potentially prevent complications such as cognitive decline are of growing importance. These treatments may improve cognitive performance in diabetes participants by addressing both vascular and neurodegenerative complications or through direct drug properties such as anti-inflammatory effects. Clinical trials have shown that improved glycaemic control can lead to improvements on self-reported and objective measures of cognitive functioning [5–7].

Metformin is the most commonly used OHA among diabetes patients and its mechanisms are to reduce hepatic glucose output and increase insulin mediated utilization of glucose in the periphery [8, 9], to restore insulin signalling pathways, and to improve insulin sensitivity in peripheral tissues [10]. Decreasing insulin resistance, normalising plasma glucose level, reducing adiposity, and reducing the formation of atherosclerotic plaques are some of mechanisms which have been proposed to explain the link between metformin and protection against neurodegeneration. Apart from peripheral effects, previous studies have also demonstrated that metformin has anti-inflammatory and neuroprotective effects on the brain [9].

Exogenous insulin is used as a treatment for type 2 diabetes patients when hyperglycaemia is uncontrolled by OHAs. As a hormone, insulin has a number of important functions in the central nervous system in a healthy human body. Thus, reduced insulin levels and insulin activity may contribute to a number of pathological processes that characterize Alzheimer's disease (AD) such as synaptic loss, limited dendritic arborisation, and memory impairment [11]. There are, however, controversial reports in the literature on the effect of insulin alone or in combination with OHA on cognitive function in diabetes. Several molecular mechanisms have been proposed regarding insulin and its protective effects (glycaemic control) and risks (anabolic effect including weight gain, inhibition of lipolysis, and enhanced lipogenesis) on cognition in patients with diabetes [12].

Though many studies have examined diabetes in relation to cognitive function, very few studies have evaluated the influence of type of diabetes treatment on the association with multiple cognitive domains in longitudinal cohort studies. The aim of the present study is to examine the relationship between type of diabetes treatment on measures of specific cognitive domains at baseline and cognitive decline over 4 years.

## 2. Research Design and Methods

### 2.1. Study Design: PATH Through Life Study

The PATH Through Life is a longitudinal cohort study of 7485 adults randomly sampled from the electoral roll of the Australian Capital Territory and Queanbeyan. This study comprises three cohorts, young (aged 20–24 years at baseline/Wave 1), midlife (aged 40–44 years at baseline/Wave 1), and older (aged 60–64 years at baseline/Wave 1). All three cohorts were followed up twice at 4 yearly intervals.

### 2.2. Participants

The present study focuses on the older aged cohort between the first follow-up in 2005-2006 (Wave 2, aged 65–69 years; *n* = 2222) and the second follow-up in 2009-2010 (Wave 3, aged 69−72 years; *n* = 1973). Participants were excluded if they reported a history of stroke (*n* = 61) and epilepsy (*n* = 14), had dementia during assessment (*n* = 1), or were on DPP 4 inhibitors or glitazones (*n* = 33) and only on insulin (*n* = 14) or if diabetes treatment data was missing (36) leaving a final sample of 1814. Details of study design and participants have been described previously [13]. The Australian National University Ethics Committee approved the study and all participants gave written informed consent to be included in this study and for their data to be linked to Australian Government Pharmaceutical Benefit Scheme (PBS) data. PBS data on dispensed medications was available only for first follow-up (Wave 2, treated as baseline in this study) and second follow-up (Wave 3, treated as follow-up) as data linkage was only established after first follow-up.

### 2.3. Measures of Type of Diabetes Treatment

At baseline and at each follow-up participants were asked to indicate if they were taking diabetes treatment (yes/no) for the following: diet and exercise, tablets, and insulin. The PATH dataset is also linked to the Australian Government Pharmaceutical Benefit Scheme (PBS) database which records all prescription medications dispensed in Australia. Data for use of oral hypoglycemic agents (OHAs) and insulin and duration of medication use were extracted from the PBS dataset and linked to PATH. We used information regarding participants' medication use from two years before the date of first follow-up interview to the date of the second follow-up interview. Thus, the variable classifying type of diabetes treatment included the data from PBS database (for those on pharmaceutical treatments) as well as self-report data for diabetes management through life style modification (diet and exercise). This methodology follows that of other published studies.

### 2.4. Measurement of Covariates

Multiple risk factors for cognitive decline that have also been linked to diabetes were selected as covariates. These included demographic factors (age, sex, and years of education) and cardiovascular factors (hypertension, smoking, physical activity, and obesity). Hypertension was defined as a measured systolic blood pressure ≥140 mmHg, diastolic blood pressure ≥90 mmHg, or self-report of antihypertensive treatment. Smoking status was obtained from a self-report questionnaire and coded into the following categories: current smoker, past smoker, and never smoked. Physical activity was assessed using the UK Whitehall II study questionnaire and coded into the following categories: mild, moderate, and vigorous activity [14]. Anthropometric measures including self-reported height (in meters) and weight (in kilograms) at each assessment were used to calculate Body Mass Index (BMI) (kg/m^2^).

### 2.5. Assessment of Cognitive Function

*Immediate Recall (first list of California Verbal Learning Test)* was used to measure verbal short-term memory [15].* Wechsler Memory Scale-Digit Span Backward* was used to test working memory [16].* Spot-the-Word (STW) Task *was used to assess verbal ability [17].* Symbol-Digit Modalities Test (SDMT)* was used to assess speed of information processing [18].* Simple Reaction Time (SRT) and Choice Reaction Time (CRT)* were also performed to measure psychomotor speed and information processing speed. Details are described elsewhere [19].* Trail Making Test, part A and part B, *provided measures of processing speed and executive function (task switching) [20]. For both tests (TMT-A and TMT-B) completion time was recorded.* Purdue Pegboard Test (both hands) (PPEG test)* was used as a measure of psychomotor speed [21].

### 2.6. Statistical Analysis

Statistical analysis was performed using the IBM SPSS (Version 22). The association between type of diabetes treatment and performance on each of the nine different neuropsychological tests was assessed using a series of Generalized Linear Models (GLZM). First cross-sectional analysis was conducted to assess the effect of type of diabetes medication on cognitive function at baseline. Participants who were diagnosed with diabetes or on diabetes treatment during baseline assessment were included in diabetes treatment groups for this analysis.

Then longitudinal analysis was conducted to assess effect of type of diabetes medication on change of cognitive function over 4 years. Model 1 adjusted only for the demographic variables (age, sex, and years of education) and Model 2 additionally adjusted for selected cardiovascular risk factors: physical activity, smoking, BMI, and hypertension. In longitudinal analysis, we compared diabetes treatment groups with no diabetes participants in total sample and compared pharmacological treatment groups to life style modification only group in diabetes only cohort. Two statistical models were used as described above using the same covariates. Participants who were diagnosed with diabetes or on diabetes treatment during baseline assessment and during follow-up were included into diabetes treatment groups. Statistical significance was kept at 0.01 to minimize type 1 error due to multiple comparisons.

## 3. Results

### 3.1. Population Characteristics

Table 1 describes characteristics of the study population at baseline according to diabetes treatment type. Participants with diabetes were more likely to be obese regardless of type of treatment compared to participants without diabetes. The percentage of participants with hypertension was higher in the diabetes group and highest in the OHA group. Participants on pharmacological treatments for diabetes were less physically active compared to no diabetes and diet only groups.

At baseline 133 participants were diagnosed with diabetes. 78 participants developed diabetes during follow-up. In this cohort we exclude participants who were only on insulin (*n* = 14), DPP 4 inhibitors, or glitazone (*n* = 33) due to sample size for each group and no consistent use. There were no participants on acarbose or byetta in this cohort. Therefore, participants on OHA are on either SU or metformin.

Participants who reported that they were on OHA and took OHA according to PBS data were categorised into the OHA group. Participants who reported that they were on diet control and whose PBS data did not indicate use of diabetes medication were allocated to the diet control only group. Participants on insulin with* or *without OHA according to PBS data were allocated to the insulin treatment group.

### 3.2. Association between Type of Diabetes Treatment and Cognitive Function

Table 2 presents mean values for individual cognitive test according to treatment group at baseline and follow-up. When comparing the baseline cognitive function with no diabetes group, there was no statistical difference between treatment groups and no diabetes group. When analysis was limited to diabetes only group, the comparison was made against life style modification group and we did not identify significant protective effect from OHA or insulin at baseline.

Then analysis was conducted to examine the effect of diabetes treatment on cognitive decline over time. There was no significant effect of type of diabetes treatment on cognitive function over 4 years compared to diet only group or compared to no diabetes group.  Table 3 presents results from the Generalized Linear Models assessing the association between type of diabetes treatment and cognitive function (cross-sectional analysis and longitudinal analysis compared to life style modification group).

### 3.3. Relation between Metformin and Cognitive Function

When comparing participants taking metformin only (*n* = 23) to those on other forms of treatment (diet, metformin with other OHAs or insulin, other OHAs, and insulin or in combination), metformin treatment alone was associated with significantly higher performance for Immediate Recall (verbal memory), Digit Span Backward (working memory), and Trail Making Test part B (executive function) at baseline. These findings did not change after adjusting for level of physical activity, smoking, BMI, and hypertension. Results for all other tests also showed higher performance to be associated with the metformin only treatment group, although these results were not statistically significant. In the longitudinal analysis, participants only on metformin (*n* = 76) showed significant protective effect only on performance for choice reaction time. Interestingly, findings for other neurological tests were also in the same direction but there was no statistical difference.  Table 4 presents findings from GLM analysis for metformin only treatment group.

In this sample, there are 49 participants on metformin and 26 of them took other OHAs or insulin in addition to metformin. We conducted a similar analysis to compare the subjects on metformin (*n* = 49) to participants who are on other forms of diabetic medications. In cross-sectional analysis, the Digit Span Backward test showed significantly better performance in the metformin group. No association was observed between metformin treatment and change of cognitive function over 4 years.

## 4. Discussion

In this study, we did not observe significant effect from OHA or OHA with insulin on cognitive function at baseline or change of cognitive function over 4 years. In this analysis, participants who used metformin to treat their diabetes appeared to have better cognitive function at baseline compared to those who used other forms of treatment. This effect was strongest for the domains of verbal memory, working memory, and executive function. We also noted significant protective effect from metformin on performance for psychomotor speed over 4 years.

Previous studies have found limited evidence for an effect of type of diabetes treatment on cognitive function. Two longitudinal studies surveying exclusively women observed similar scores on cognitive function tests (TICS and a global score) for participants without diabetes and for participants in OHA treatment diabetes groups [22, 23]. Another report from an Australian study observed greater decline in executive function and global cognition in participants with diabetes. However, in further analysis, they found no relationship between treatment types (OHA versus diet control) and decline in global cognitive function [24]. In contrast, Elias et al. [25] reported poor cognitive performance (Wechsler Logical Memory story recall) among participants with diabetes but found that compared to healthy controls the insulin treatment group performed more poorly on cognitive measures (immediate and delayed verbal memory on the story recall test and visual memory). Interestingly, performance for participants who were treated with oral agents did not differ from the no diabetes reference group on any of the 7 neuropsychological tests. A report from Women's Health Initiative study reported better verbal knowledge among participants treated with oral medications and reduced psychomotor speed in those using insulin. This study did not, however, find an effect of type of diabetes treatment on cognitive decline [26]. Moore et al. examined the effect of diabetes and of metformin treatment on cognitive function in the Primary Research in Memory (PRIME) study and the Australian Imaging, Biomarkers, and Lifestyle (AIBL) study. Among participants with diabetes, worse cognitive performance was associated with metformin use (*n* = 35) (after controlling for age, sex, depression, and level of education). This study used only MMSE as a measure of cognitive function and did not control for other treatments used in conjunction with metformin when analyzing the association with metformin. This association was not significant after they adjusted their analysis for serum B12 level [27]. Ng et al. [28]. (2014) also examined effect of use of metformin (*n* = 365) on cognitive function using MMSE in a relatively larger cohort study. They observed a significant protective effect of using metformin in both cross-sectional and longitudinal analyses. This is the only study to date that has adjusted their analysis for glycaemic control. To the best of authors' knowledge there are no other studies that have examined the effect of metformin on level of cognitive function from a longitudinal cohort study. However, in both the above studies, participants in the metformin group may have been using other treatments in conjunction with metformin. Together with the available evidence, our findings also suggest that metformin may have a protective effect on cognitive function when compared to other treatments.

The present study has some limitations. Even though we adjusted models for multiple confounding factors we were unable to adjust models for other important factors for which measures were not available, including glycaemic control, plasma insulin level, type of diabetes, duration of diabetes, and level of renal function. As an example, metformin is first-line medication for diabetes and is used commonly when someone has uncomplicated diabetes. Metformin is contraindicated in advanced kidney disease. Diabetes with complications needs more than one OHA with or without insulin to maintain glycaemic control. Level of glycaemic control and diabetes complications could directly impact on the level of cognitive function. Therefore the results need to be interpreted with caution. The PBS data only contains data for medications dispensed to individuals and adherence to these medications is not available. We also did not have data on other factors relating to treatment type (such as willingness to take medication) and whether diet control was used alongside OHA and insulin; hence the effect of diet control on cognition could not be assessed reliably. When we compared the different types of treatment groups (diet, OHA, and insulin) sample size for each group was small, thus reducing statistical power. The duration of follow-up in the present study is relatively short and this may influence our findings as the symptoms of cognitive disease have long duration. The percentage of participants with diabetes (11.6%) in this cohort is slightly less than Australian prevalence rates. According to the Australian Institute of Health and Welfare (2007-2008) 14% of the general population aged between 64 and 70 years were diagnosed with diabetes. Therefore, it is possible that undiagnosed diabetes participants were included into no diabetes group.

This study also has several strengths. First the analyses include data from a large longitudinal cohort. The PATH Through Life project also provides a unique opportunity to examine multiple cognitive domains. We were also able to use a more objective measure of medication use (PBS data) than self-report as has been used in many prior studies. Furthermore, a large number of covariates were included in the analysis. This enabled adjustment for a broad array of confounders. Most importantly, to the best of our knowledge this is the first study that has examined the effect of metformin on multiple cognitive domains in a longitudinal cohort study.

In summary, we did not find any association between types of diabetes treatment with cognitive function. In regard to therapeutic risk assessment, metformin appears to provide some protection against cognitive function although the exact mechanisms are unclear and this effect could also be attributable at least in part to other factors differentiating the treatment groups. Therefore, adequately powered RCTs are needed to establish long-term effects of antidiabetic medications on cognition.

## Figures and Tables

**Table 1:** Population characteristics according to type of diabetes treatment.

	No diabetes (1603)	Diabetes (211)		
		Diet control (74)	OHA only (113)	Insulin (24)
Age (mean (SD))	66.6 ± 1.5	66.3 ± 1.5	66.7 ± 1.6	67.3 ± .3
Males (*N* (%))	818 (51.0)	35 (47.3)	63 (55.8)	12 (50.0)
Years of education (mean (SD))	14.1 ± 2.7	14.0 ± 2.8	13.1 ± 2.6	14.1 ± 2.6
BMI (mean (SD))	26.4 ± 4.6	29.8 ± 6.2	29.7 ± 5.3	30.4 ± 5.8
Hypertension (*N* (%))	1001 (62.8)	60 (82.2)	99 (87.6)	18 (75.0)
Physical activity (*N* (%))				
Mild	522 (33.7)	28 (38.4)	45 (41.3)	11 (52.4)
Moderate	783 (50.5)	34 (46.6)	56 (51.4)	10 (47.6)
Vigorous	246 (15.9)	11 (15.1)	8 (7.3)	0 (0)
Smoking (*N* (%))				
Never	914 (57.1)	27 (36.5)	48 (42.5)	10 (41.7)
Past	573 (35.8)	40 (54.1)	59 (52.2)	13 (54.2)
Current	115 (7.2)	7 (9.5)	6 (5.3)	10 (4.2)

**Table 2:** Association between type of diabetes treatment and cognitive function (mean and SD).

	MMSE	SDMT	Imm. Rec.	STW	Digit Back.	Trail A	Trail B	PPEG (both hands)	SRT	CRT
No diabetes (*n* = 1603)										
Baseline	29.3 (1.2)	50.3 (9.0)	7.1 (2.2)	53.2 (5.2)	5.3 (2.2)	33.9 (10.6)	78.0 (30.4)	10.1 (1.8)	.3 (.1)	.3 (.1)
Follow-up	29.1 (1.3)	48.3 (9.1)	6.7 (2.3)	53.3 (5.0)	5.1 (2.2)	35.9 (13.2)	83.3 (33.6)	9.5 (1.8)	.3 (.1)	.3 (.1)
Change	−.2	−2.0	−.4	.1	−.2	2.0	5.3	−.6	0	0
Diet only (*n* = 74)										
Baseline	29.1 (1.1)	49.1 (8.1)	6.8 (1.9)	53.8 (4.6)	5.0 (2.3)	33.9 (11.0)	79.3 (27.3)	10.0 (1.7)	.3 (.1)	.4 (.1)
Follow-up	29.2 (1.0)	45.6 (9.3)	6.1 (2.2)	53.6 (5.0)	4.7 (1.9)	39.9 (22.9)	85.4 (29.5)	9.2 (1.7)	.3 (.1)	.3 (.1)
Change	.1	−3.5	−.7	−.2	−.3	6.0	6.1	−.8	0	−.1
OHA only (*n* = 113)										
Baseline	29.1 (1.2)	46.8 (9.6)	6.4 (2.6)	52.5 (5.6)	4.8 (1.9)	36.0 (12.6)	85.6 (37.4)	10.1 (1.9)	.3 (.1)	.3 (.1)
Follow-up	29.1 (1.1)	44.4 (9.9)	6.5 (2.2)	52.4 (5.5)	4.7 (2.1)	40.1 (16.1)	95.7 (37.0)	9.1 (1.9)	.3 (.1)	.3 (.1)
Change	0	−2.4	.1	−.1	−.1	4.1	10.1	−1.0	0	0
OHA + ins. (*n* = 24)										
Baseline	29.2 (1.2)	47.5 (7.5)	5.5 (2.0)	53.4 (4.4)	3.6 (2.4)	42.9 (15.3)	85.2 (24.8)	8.8 (2.2)	.3 (.1)	.4 (.1)
Follow-up	29.0 (.9)	43.4 (9.0)	5.9 (2.8)	53.2 (4.7)	4.7 (2.1)	46.2 (20.0)	102.1 (39.5)	7.7 (2.1)	.3 (.1)	.4 (.1)
Change	−.2	−4.1	.4	−.2	1.1	3.3	16.9	−1.1	0	0

Note: measures for Trail A, Trail B, SRT, and CRT represent response time. Thus, positive values for change indicate cognitive decline. All other measures (SDMT, Imm. Rec., STW, Digit Back., and PPEG (both hands)) represent number of items completed correctly (negative values for change indicate cognitive decline).

**Table 3:** Association between type of diabetes treatment and cognitive function (*β* weights and SE).

	SDMT	Imm. Rec.	STW	Digit Back.	Trail A	Trail B	PPEG (both hands)	SRT	CRT
Cross-sectional									
M1-OHA only	.14 (2.01)	.61 (.70)	−2.19 (.99)	−.04 (.59)	−2.18 (2.66)	3.67 (7.99)	.28 (.38)	−.02 (.01)	−.00 (.01)
OHA + insulin	−1.02 (2.88)	−.68 (.70)	−4.02 (1.39)	.70 (.42)	4.12 (3.79)	16.92 (11.31)	−.12 (.54)	.01 (.01)	.03 (.01)
M2-OHA only	−.46 (2.08)	.75 (.47)	−2.28 (1.01)	.57 (.45)	−2.76 (2.72)	−.99 (6.62)	.37 (.40)	−.02 (.01)	−.02 (.02)
OHA + insulin	−.98 (3.01)	−.46 (.67)	−4.39 (1.46)	−.12 (.64)	3.60 (3.94)	15.74 (9.56)	−.12 (.58)	−.01 (.02)	−.02 (.02)
Longitudinal									
M1-OHA only	1.15 (.84)	.51 (.30)	.03 (.34)	.31 (.24)	−5.20 (3.48)	11.81 (4.63)	−.10 (.23)	−.01 (.01)	−.01 (.01)
OHA + insulin	−.3 (1.25)	.16 (.44)	−.12 (.51)	.30 (.35)	−3.37 (3.85)	11.81 (6.81)	−.76 (.35)	.01 (.01)	.00 (.01)
M2-OHA only	.66 (1.36)	.47 (.50)	−.04 (.53)	.17 (.38)	−6.38 (4.12)	3.36 (7.11)	−.53 (.35)	−.02 (.01)	−.04 (.01)
OHA + insulin	−1.97 (1.91)	.27 (.70)	−.38 (.75)	.04 (.53)	−9.43 (5.88)	16.14 (9.97)	−.94 (.550)	−.00 (.02)	−.03 (.02)

Note: measures for Trail A, Trail B, SRT, and CRT represent response time. Thus, positive *β* values indicate poorer performance relative to no diabetes group. All other measures (MMSE, SDMT, Imm. Rec., STW, Digit Back., and PPEG (both hands)) represent number of items completed correctly (negative *β* values indicate poorer performance).

Model 1 = control for age, sex, and education; W2 cognitive function.

Model 2 = Model 1 + BMI, PA, smoking, and hypertension.

**Table 4:** Association between type of diabetes treatment and cognitive function (*β* weights and SE).

	MMSE	SDMT	Imm. Rec.	STW	Digit Back.	Trail A	Trail B	PPEG (both hands)	SRT	CRT
Cross-sectional										
M1-met. only	.03 (.16)	2.04 (2.21)	1.26 (.54)^*∗*^	.24 (1.15)	1.14 (.45)^*∗*^	−3.47 (2.47)	−17.76 (8.68)^*∗*^	−.04 (.42)	−.00 (.02)	−.00 (.01)
M2-met. only	.03 (.15)	2.11 (2.25)	1.32 (.50)^*∗∗*^	.04 (1.16)	1.17 (.47)^*∗*^	−3.72 (2.98)	−14.42 (7.06)^*∗*^	.14 (.44)	−.01 (.02)	−.01 (.01)
Longitudinal										
M1-met. only	−.10 (.16)	1.06 (1.47)	.24 (.36)	−.33 (.73)	.10 (.30)	−3.00 (3.04)	−.70 (5.57)	.48 (.30)	−.02 (.01)	−.03 (.01)^*∗*^
M2-met. only	−.04 (.15)	1.21 (.81)	.30 (.29)	.09 (.32)	.43 (.60)	−2.72 (6.47)	−5.81 (12.10)	−.08 (.56)	−.01 (.02)	−.03 (.02)

Note: measures for Trail A, Trail B, SRT, and CRT represent response time. Thus, positive *β* values indicate poorer performance relative to no diabetes group. All other measures (MMSE, SDMT, Imm. Rec., STW, Digit Back., and PPEG (both hands)) represent number of items completed correctly (negative *β* values indicate poorer performance).

^*∗*^ *p* < 0.01, ^*∗∗*^ *p* < 0.001.

Model 1 = control for age, sex, and education; W2 cognitive function.

Model 2 = Model 1 + BMI, PA, smoking, and hypertension.
